# Blood Pressure Monitoring System Using a Two-Channel Ballistocardiogram and Convolutional Neural Networks

**DOI:** 10.3390/s21072303

**Published:** 2021-03-25

**Authors:** Woojoon Seok, Kwang Jin Lee, Dongrae Cho, Jongryun Roh, Sayup Kim

**Affiliations:** 1 Human Convergence Technology R&D Department, Korea Institute of Industrial Technology, 143 Hanggaulro, Ansan 15588, Korea; swj@deep-medi.com (W.S.); ssaccn@kitech.re.kr (J.R.); 2 Deep Medi Research Institute of Technology, Deep Medi Inc., Seoul 06232, Korea; kjlee@deep-medi.com (K.J.L.); dongrae30@deep-medi.com (D.C.)

**Keywords:** cuffless blood pressure monitoring system, hypertension, ballistocardiogram (BCG), convolutional neural network (CNN)

## Abstract

Hypertension is a chronic disease that kills 7.6 million people worldwide annually. A continuous blood pressure monitoring system is required to accurately diagnose hypertension. Here, a chair-shaped ballistocardiogram (BCG)-based blood pressure estimation system was developed with no sensors attached to users. Two experimental sessions were conducted with 30 subjects. In the first session, two-channel BCG and blood pressure data were recorded for each subject. In the second session, the two-channel BCG and blood pressure data were recorded after running on a treadmill and then resting on the newly developed system. The empirical mode decomposition algorithm was used to remove noise in the two-channel BCG, and the instantaneous phase was calculated by applying a Hilbert transform to the first intrinsic mode functions. After training a convolutional neural network regression model that predicts the systolic and diastolic blood pressures (SBP and DBP) from the two-channel BCG phase, the results of the first session (rest) and second session (recovery) were compared. The results confirmed that the proposed model accurately estimates the rapidly rising blood pressure in the recovery state. Results from the rest sessions satisfied the Association for the Advancement of Medical Instrumentation (AAMI) international standards. The standard deviation of the SBP results in the recovery session exceeded 0.7.

## 1. Introduction

Hypertension is a chronic disease that kills 7.6 million people worldwide every year [1]. It is challenging to accurately diagnose this disease due to masked hypertension, white coat hypertension, and nocturnal hypertension [2,3]. Continuous blood pressure (BP) monitoring systems are required to accurately diagnose hypertension to help prevent various diseases [4]. Ambulatory BP monitoring has been found to be more helpful than clinical BP measurements in predicting various cardiovascular diseases [5].

Most methods to measure BP in non-clinical settings, including ambulatory BP monitoring, require a cuff to be worn around the upper arm; however, because this method is inconvenient, many people fail to measure their BP regularly. A non-intrusive BP monitoring system and BP estimation technologies were developed to replace the conventional cuff method [6].

The most frequently used cuffless methods of estimating BP use electrocardiograms (ECGs), photoplethysmograms (PPGs) [7], or dual PPG [8] signals. The pulse transit time (PTT) and pulse wave velocity (PWV) extracted from these signals are known to be highly correlated with BP [4,9]. Conventional methods of measuring PPG and ECG signals attach sensors to fingers (PPG) or electrodes to the body (ECG). However, many non-intrusive signal measurement methods have been developed recently, providing a convenient method for users to measure signals [10,11]. Ballistocardiogram (BCG) signals are bio-signals, which can be used to determine the heart rate and respiratory rate of users non-intrusively [12]. BCG signals are difficult to analyze because they have non-linear and non-stationary characteristics; however, the BCG-based PTTs are highly correlated with BP [12,13].

It is important to estimate the normal BP, but it is also essential to make accurate estimates even during rapid increases in BP due to factors such as exercise and stress. In a previous study [14], we estimated subjects’ BP using the instantaneous phase difference (IPD) extracted from the BCG signals. In this study, to examine the performance of the proposed model for estimating the BPs of subjects during rapid increases, we induced an increase in the BP of the subjects and measured their BP until it returned to normal. There are two major differences between this study and the previous one. First, the BP of the subjects was increased rapidly by having them run on a treadmill, which showed a significant difference from the BP measured in the previous study. Second, whereas the subjects’ BP in the previous study was estimated using the IPD and calculated from the BCG, in this study, the BCG phase was calculated, and the BP was estimated using a convolutional neural network (CNN) regression model.

Related studies on BP estimation extracted ECG, PPG, and BCG peaks and measured the distance between the peaks to calculate the PTT and PWV to estimate BP. One disadvantage of this PTT based method is that if the peak detection is not performed correctly, the BP estimation performance may decrease. This method is highly feature-dependent, as the BP estimation model performs a regression analysis on the previously extracted features rather than learning the patterns in the signals. In our previous study, we also determined the IPD by calculating the difference between the phases of the two-channel BCG.

To overcome the drawbacks of feature-based methods, deep learning methods are frequently utilized. An increasing number of studies have created an end-to-end model by applying deep learning to analyze bio-signals, such as EEG, ECG, PPG, and BCG, instead of manually extracting features. In a related study, CNNs and gated recurrent units (GRUs) were utilized for bio-signal analysis, which resulted in superior feature-based methods [15,16].

In the present study, a CNN was used to improve upon the currently available feature-dependent methods. We propose a CNN regression model that learns the patterns within the signal of the two-channel BCG phase without extracting the IPD.

## 2. Materials and Methods

### 2.1. System Summary

**Figure 1:**
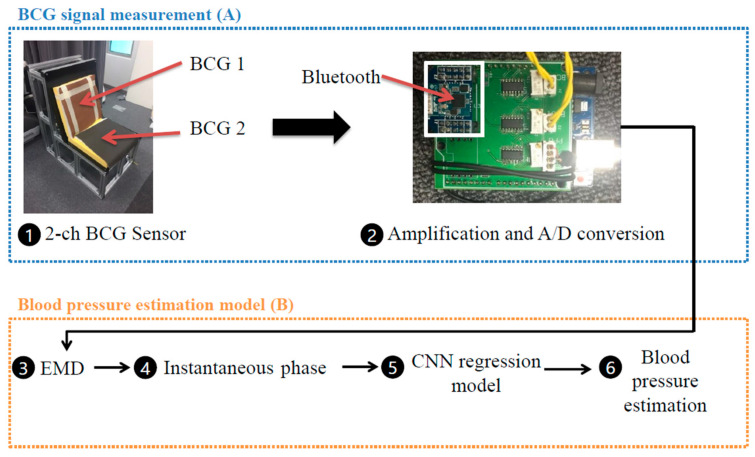
The overall workflow of the chair-shaped ballistocardiogram (BCG)-based blood pressure estimation system; (**A**) BCG signal measurement; (**B**) Blood pressure estimation model.

A sofa-style experimental chair was fabricated to provide an environment where the BCG of the subjects could be measured while they are seated freely. The seat was constructed by inserting a polyvinylidene fluoride resin (PVDF) film between the cushions of the chair and natural leather to measure the BCG signals from the subject’s back and thighs. A PPG sensor (RP520, Laxtha, Daejeon, Korea) attached to the subject’s fingers was designed to take measurements simultaneously with the BCG sensor. The PPG and BCG signals were sent from a proprietary board with a sampling frequency of 100 Hz, using the Atmega256 MCU (Atmel Corporation, San Jose, CA, USA). The BCG signals were transmitted wirelessly to a computer via Bluetooth (Parani ESD-200, Sena Technologies, Seoul, Korea) (Figure 1A). Noise removal, preprocessing of the PPG and BCG signals, and creation of the BP estimation model were performed in Python 3.7.1 (Python Software Foundation, Fredericksburg, VA, USA) [17] and Tensorflow 2.3.1 (Google, Mountain View, CA, USA) [18] (Figure 1B). The overall system structure is presented in Figure 1.

### 2.2. Experimental Procedure

The experiment in this study was conducted with a total of 30 subjects (14 males and 16 females), including subjects from a wide range of ages (20–50s). Patient averages were as follows: age: 35.3 ± 12.5 years, height: 166.1 ± 9.4 cm, weight: 63.3 ± 12.8 kg. Healthy subjects without hypertension were selected for this study. This study was approved by the Public Organization Bioethics Committee designated by the Ministry of Health and Welfare of South Korea (IRB P01-2018012-11-001).

All subjects started the experiment at 2 PM, and the experiment consisted of two sessions. In the first session (rest session), the reference BP was measured with a cuff-type BP gauge (HEM-7121, Omron, Kyoto, Japan), and the BCG signals were simultaneously measured five times per minute. In the second session (recovery session), the subjects ran on a treadmill (Radon, Drax, Anyang, Korea) where the speed and slope were electronically controlled based on the Balke incremental treadmill test [19]. The subject’s maximum heart rate was calculated as “220-age,” and the treadmill was stopped when 80% of their maximum heart rate was reached [20]. The average exercise intensity given to the subjects was 79.5 ± 8.2%. After running on the treadmill, the subjects rested by sitting on the chair-shaped blood pressure estimation system. During recovery, their BP and heart rate were measured 10 times per minute with the chair-shaped blood pressure estimation system and the cuff-type blood pressure gauge simultaneously.

### 2.3. BCG Signal Processing Using Empirical Mode Decomposition

**Figure 2:**
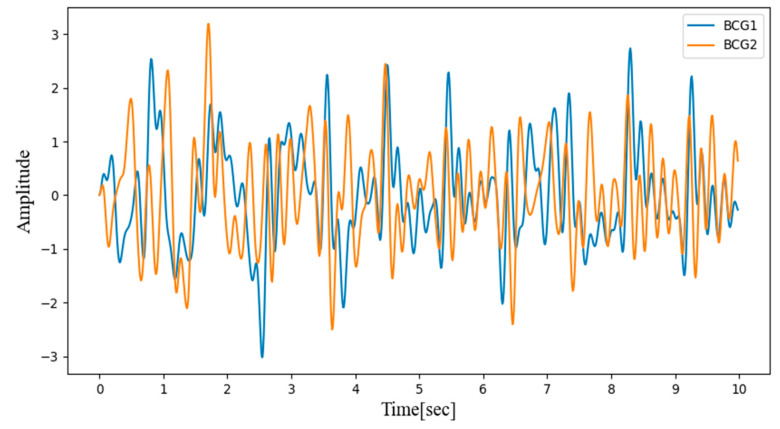
Two-channel BCG signals measured in the back and bottom seat applied to the Butterworth band-pass filter.

The BCG signals contain diverse health information such as heart rate, respiratory rate, and body movements. A third-order Butterworth band-pass filter was used to isolate the heart rate signals, with the cut-off frequency set between 0.5 Hz and 6 Hz. Figure 2 shows the results after filtering the two-channel BCG signals measured in the back and bottom seat.

Even when a band-pass filter is applied, it is challenging to effectively extract cardiorespiratory signals from the BCG signals. Empirical mode decomposition (EMD) is generally utilized to handle BCG signals and is more appropriate than other signal process algorithms. Therefore, an EMD algorithm was used to preprocess the BCG signals in this study. The process of decomposing the signals using the EMD algorithm is called the “sifting process.” The intrinsic mode functions (IMFs) are extracted from the signals containing high-frequency domains, and IMF (1), IMF (2), …IMF (n) are extracted, where n is the total number of IMFs. The EMD algorithm is constructed as follows [21]:

1. Find the maximum and minimum values of the raw signals x(t) and create upper and lower envelopes using spline interpolation.
2. Calculate the difference between the upper and lower envelopes, and estimate the average m(t) of the maximum and minimum envelopes.
3. Extract new signals *h_n_*(t), which is the raw signal x(t) minus the average m(t), that become each IMF (n). Repeat steps 1–3.
4. Stop repeating when the new IMF signal has only a single extreme value and is expressed as a monotone function.

**Figure 3:**
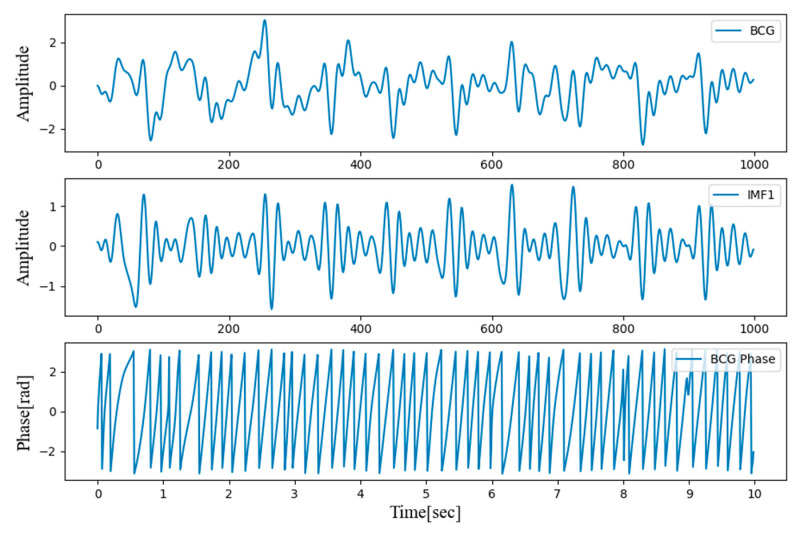
Filtered BCG signal, first intrinsic mode function (IMF) extracted using the empirical mode decomposition (EMD) algorithm, and BCG phase calculated by Hilbert Transform.

The instantaneous phase was obtained by applying a Hilbert transform to the first IMF extracted by the EMD algorithm. The resulting instantaneous phase is presented in Figure 3.

### 2.4. 1-D CNN

A CNN regression model was implemented to estimate subjects’ BP. CNNs learn the BCG signal patterns by utilizing the instantaneous phase, calculated from the two-channel BCG, as an input. Our previous study performed a regression analysis with an artificial neural network (ANN) using the IPD calculated from the differences in the two-channel BCG phase. However, in this study, the CNN model learned the pattern of the two-channel BCG phase and hence did not require IPD features.

**Figure 4:**
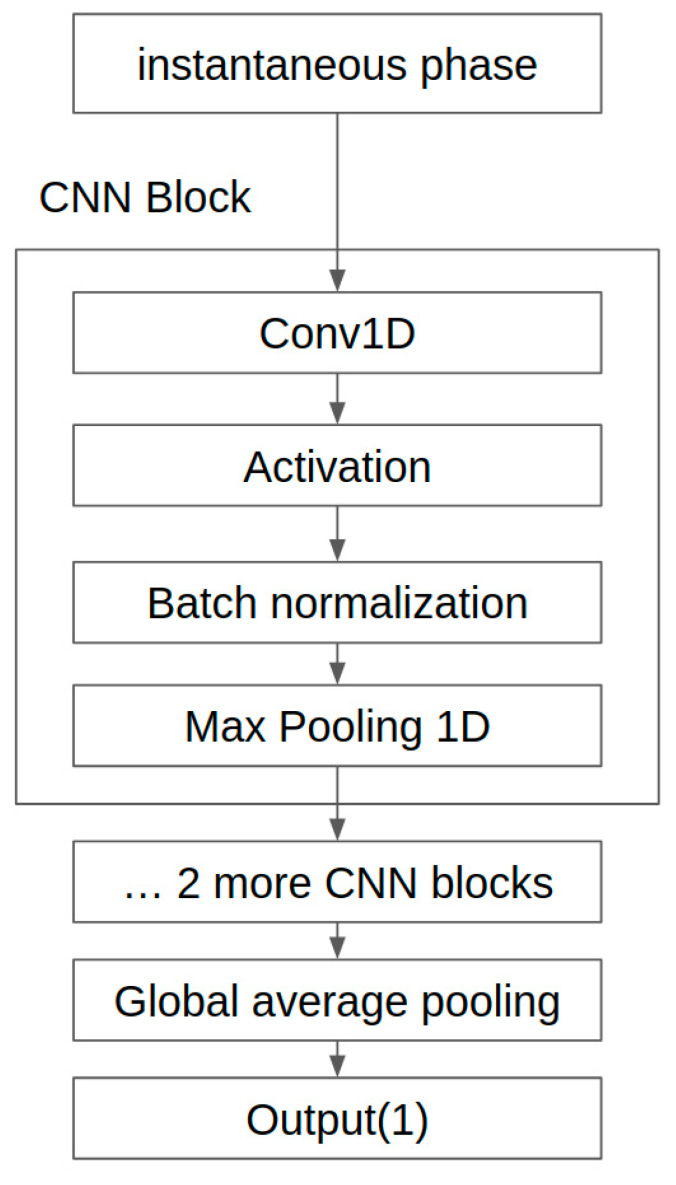
The architecture of the convolutional neural network (CNN) model.

The CNN model was implemented using Python 3.7.1 and Tensorflow 2.3.1. A single CNN model was implemented to estimate the systolic and diastolic blood pressures (SBP and DBP) in both the rest and recovery sessions. The architecture of the proposed CNN model is presented in Figure 4.

The input of the CNN regression model takes two channel BCG phases. The CNN regression model estimated the BP with two 10 s channel BCG phases and was trained separately to estimate the SBP and DBP.

The CNN model comprises three convolution layers, max-pooling layers, one global average pooling layer, and one dense layer. The first 1-D convolution layer has 100 filters, with a kernel size of 21 and one stride. Since the number of filters is usually a multiple of the sampling rate when bio-signals such as PPG and ECG are applied to the 1-D CNN, it was set to the BCG sampling frequency, that is, 100 Hz [22]. The second and third 1-D convolution layers were set to 200 Hz and 300 Hz by multiplying the BCG sampling frequency by two and three, respectively. The kernel size was decreased from 21 in the first convolutional layer to five in the second and third layers. The input of each layer was normalized through the batch normalization layer after passing through the convolutional layer. Then nonlinearity was added using the rectified linear unit (ReLU) activation function. The max-pooling layers were placed after the first and second convolutional layers, which decreases the sequence length through the CNN by half. After the final convolutional layer, the global average pooling layer was replaced with the used flattened layer [23]. Only one layer was used for the final dense layer because overfitting occurred when composed of multiple fully connected layers. The final layer for regression used the identity activation function, which outputs the input value as it is. This value is directly connected to BP. To train the model, the mean squared error loss was used. The learning rate was set to 0.001, and the Adam optimizer was used as the optimizer.

As the input consists of BP (SBP and DBP) and BCG signals every 10 s, a 10 s BCG signal was used as a single epoch. The BCG and BP were measured five times for 10 s in the rest session and 10 times for 10 s in the recovery session. The training and test sets were divided by an 8:2 ratio. After separation, the training set was further partitioned into 10 equally sized segments to conduct 10-fold cross-validation.

## 3. Results

### BP Estimation Model

After training with the 10-fold cross-validation, the optimal CNN regression model estimated the BPs in the test set. Table 1 lists the mean error (ME) and standard deviation (SD) of the SBP and DBP estimations by the CNN model for the rest and recovery sessions.

As shown in Table 1, the ME and SD of the SBP and DBP in the rest sessions were lower than the National Standards Institute/Association for the Advancement of Medical Instrumentation/International Organization for Standardization (ANSI/AAMI/ISO) 2013 protocol (ME < 5 mmHg, SD < 8 mmHg) [24]. In the recovery session, the SD values slightly exceeded the AAMI standard.

**Figure 5:**
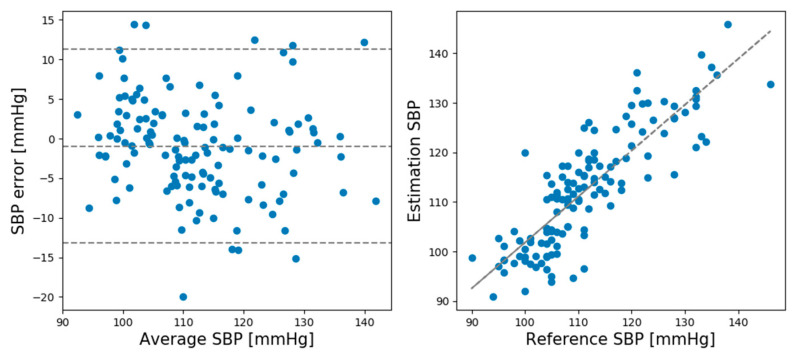
Bland–Altman plots for systolic blood pressure (SBP) estimation result of the CNN model in the rest session.

**Figure 6:**
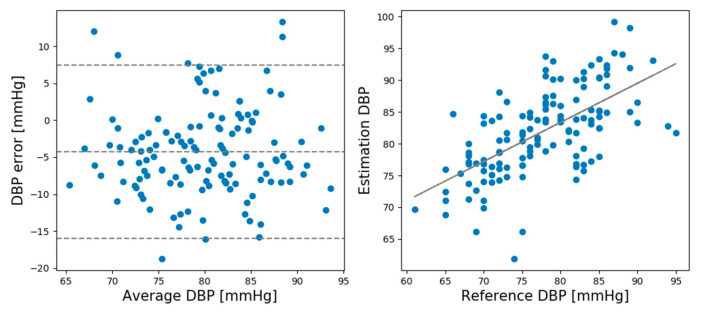
Bland–Altman plots for diastolic blood pressure (DBP) estimations made by the CNN Model in the rest session.

**Figure 7:**
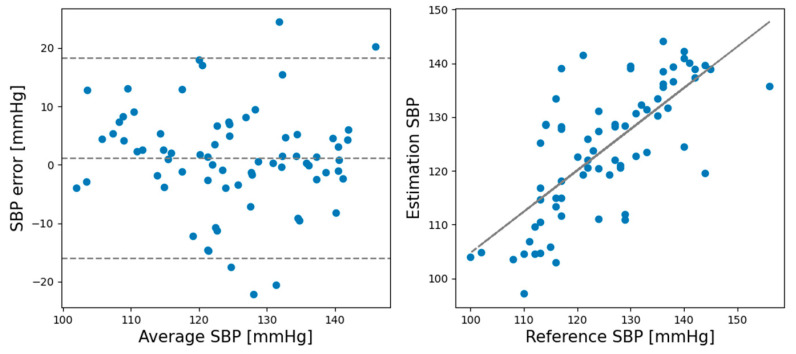
Bland–Altman plots for SBP estimations result made by CNN model in the recovery session.

**Figure 8:**
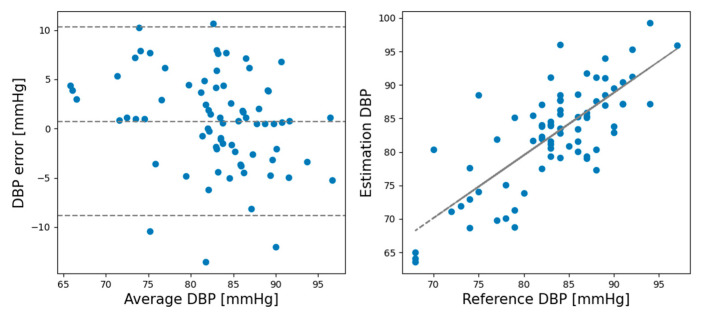
Bland–Altman plots for DBP estimations made by the CNN model in the recovery session.

In the rest session, the ME and SD of the predicted and actual BP values of the SBP prediction model were 0.93 and 6.24, respectively, while the ME and SD of the DBP prediction model were 0.21 and 5.42, respectively. In the recovery session, the ME and SD of the predicted and actual BP values of the SBP prediction model were −1.12 and 8.74, respectively, and the ME and SD of the DBP prediction model were −0.728 and 4.87, respectively. Comparing the ME and SD between the rest and recovery sessions shows that the proposed model performed better for the rest session than the recovery session except DBP standard deviation. Figure 5 shows Bland–Altman plots for the SBP estimations made by the CNN model in the rest session. Figure 6 shows the Bland–Altman plots for the DBP estimations made by the CNN model in the rest session. Figure 7 shows the Bland–Altman plots of the SBP estimations made by the CNN model in the recovery session. Figure 8 shows the Bland–Altman plots of the DBP estimations results made by the CNN model in the recovery session.

## 4. Discussion

In this study, we developed a chair-shaped BCG measurement system using two PVDF films to non-intrusively estimate the BP of users. Previous studies have developed chair-shaped blood pressure estimation systems, but they require users to keep touching the hand-rest of the chair [25]. In contrast, the system developed in this study has the advantage that BP can be measured while the user is seated in a chair.

A previous study calculated the difference between the 2-channel BCG phases using the IPD in the rest session and found that the IPD is more strongly correlated to BP than the PTT [14]. In this study, the recovery session was added to verify the system’s performance at high BPs. Instead of merely measuring the high BP, it was measured over a 10 min period while the heart rate of subjects returned to normal after exercising to 80% of their maximum heart rate.

In our previous study, BP was estimated using data from a rest session. As a result, the standard deviation was 6.74 in SBP and 5.83 in DBP. However, in the CNN model, the standard deviation was 6.24 in SBP and 5.42 in DBP. The standard deviation difference in SBP was about 0.5, and the CNN regression model result was better than that of the IPD based ANN model. The standard deviation difference in DBP was about 0.41, and the CNN model showed better results than IPD. The IPD feature is calculated when the difference between BCG 2-channel phases becomes constant. It could not be used in the recovery session due to the noise caused by the breathing and movements of subjects when they exercised. Therefore, we could not compare the IPD and the CNN regression model in the recovery session dataset. However, the CNN model learns the relationship between BCG 2-channel phases without calculating IPD. Moreover, it can predict BP in a recovery session with similar performance to a rest session.

While measuring the BP, many factors (gender, age, disease, BP measurement time, etc.) have a significant influence on the BP estimation. Therefore, by increasing the BP of the subjects through exercise, we tried to exclude the factors that interfere with BP measurement. Despite the noise in the recovery session dataset the CNN regression model exhibited good BP estimation performance. When we attempted to train the CNN model with the rest and recovery sessions together, the model exhibited overfitting. Therefore, the CNN regression model was trained separately for each session. As a result, it was possible to estimate the BP in the recovery session.

One limitation of this study is that only 30 subjects were included, which is smaller than the number of subjects (85 minimum) recommended by the AAMI for evaluating a blood pressure gauge [26]. If 85 or more subjects can be secured in additional experiments, the CNN model’s performance could be enhanced, and the BPs could be predicted more accurately. Additionally, we plan to investigate the training of raw BCG signals using deep learning, utilizing continuous BCG signals’ characteristics. We also plan to investigate a deep learning model’s training with improved better performance by creating an end-to-end model without preprocessing.

This study’s results are particularly applicable to patients with chronic diseases who are seated on sofas or chairs in houses and/or offices, dialysis patients who need constant BP checks, and even to patients in medical beds, in and out of the clinical setting. Moreover, the proposed system can measure BP with high accuracy even when the patient’s BP rises abnormally.

## Figures and Tables

**Table 1:** Mean errors (ME) and standard deviations (SD) of systolic and diastolic blood pressures (SBP and DBP) in the rest and recovery sessions.

	SBP (mmHg)		DBP (mmHg)	
	ME	SD	ME	SD
Rest	0.93	6.24	0.21	5.42
Recovery	−1.12	8.74	−0.728	4.87

## Data Availability

Not applicable.

## Abbreviations

The following abbreviations are used in this manuscript:

AAMI	Association for the Advancement of Medical Instrumentation
ANN	Artificial Neural Network
ANSI	American National Standards Institute
BCG	Ballistocardiogram
BP	Blood Pressure
CNN	Convolutional Neural Network
DBP	Diastolic blood pressure
ECG	Electrocardiogram
EMD	Empirical mode decomposition
GRU	Gated recurrent units
IMF	Intrinsic mode function
IPD	Instantaneous phase difference
ISO	International Organization for Standardization
ME	Mean error
PPG	Photoplethysmogram
PTT	Pulse transit time
PVDF	Polyvinylidene fluoride resin
PWV	Pulse wave velocity
SBP	Systolic blood pressure
SD	Standard deviation
